# An application of the ensemble Kalman filter in epidemiological modelling

**DOI:** 10.1371/journal.pone.0256227

**Published:** 2021-08-19

**Authors:** Rajnesh Lal, Weidong Huang, Zhenquan Li

**Affiliations:** 1 School of Mathematical and Computing Sciences, Fiji National University, Lautoka, Fiji; 2 TD School, University of Technology Sydney, Ultimo, NSW, Australia; 3 School of Computing and Mathematics, Charles Sturt University, Thurgoona, NSW, Australia; University of Parma: Universita degli Studi di Parma, ITALY

## Abstract

Since the novel coronavirus (COVID-19) outbreak in China, and due to the open accessibility of COVID-19 data, several researchers and modellers revisited the classical epidemiological models to evaluate their practical applicability. While mathematical compartmental models can predict various contagious viruses’ dynamics, their efficiency depends on the model parameters. Recently, several parameter estimation methods have been proposed for different models. In this study, we evaluated the Ensemble Kalman filter’s performance (EnKF) in the estimation of time-varying model parameters with synthetic data and the real COVID-19 data of Hubei province, China. Contrary to the previous works, in the current study, the effect of damping factors on an augmented EnKF is studied. An augmented EnKF algorithm is provided, and we present how the filter performs in estimating models using uncertain observational (reported) data. Results obtained confirm that the augumented-EnKF approach can provide reliable model parameter estimates. Additionally, there was a good fit of profiles between model simulation and the reported COVID-19 data confirming the possibility of using the augmented-EnKF approach for reliable model parameter estimation.

## Introduction

The outbreak of the novel coronavirus disease (COVID-19) in early December 2019 in Wuhan, China, attracted many researchers to evaluate the dynamics of infectious COVID-19 virus using various mathematical models [1–17]. Mathematical compartmental models, such as SIR (Susceptible—Infectious—Recovered) [18, 19], in epidemiology, are generally expressed by a system of ordinary differential equations (ODE). Recent studies on COVID-19 modelling includes using the basic SIR model [12, 18, 19] or its extension (modified) versions such as SEIR (Susceptible—Exposed—Infectious—Recovered) [7, 10, 11, 19–21], SIRD (Susceptible—Infectious—Recovered—Dead) [1–4, 16, 17, 22] and SEIRD (Susceptible—Exposed—Infected—Recovered—Dead) [13–15].

There are many significant practical importance of epidemiological modelling via computer simulation, which includes understanding the disease and dynamics of the infectious virus, e.g. predicting the number of new cases [23]. Also, the outcome of modelling and simulations can provide vital information to governments and decision-makers [23]. However, a mathematical model’s performance and reliability depend on uncertainties in the model and the model parameters [24]. While identifying accurate parameters of models, e.g. infection rate and mortality rates, is an essential exercise in mathematical modelling, precise prediction of parameters is a difficult task [23].

Model parameters can be estimated by solving inverse problems using observational data [24]. Some recent works on inverse COVID-19 modelling with different models include works of Wan et al. [7], Sun et al. [10], Libotte et al. [18], Lobato et al. [2], Li et al. [11], Anastassopoulou et al. [3], Loli Piccolomini and Zama [14], Korolev [15], Ndaïrou et al. [9], Ifguis et al. [12], Engbert et al. [21], Yang et al. [20], Arroyo-Marioli et al. [25], and Ghostine et al. [26]. Recent methods of inverse modelling for parameter estimation include the least-square techniques and optimization algorithms [3, 10, 12, 14, 15], Differential Evolution method [18], Stochastic and Multiobjective Fractal Search algorithm [2], and data assimilation methods [11, 20, 21, 25, 26].

Introduced by Geir Evenson [27], the ensemble Kalman filter (EnKF) is a data assimilation technique that can be employed to update both model parameters and states variables with their associated uncertainties [28]. Yang et al. [20] used the EnKF for joint state-parameter estimation, where one of the SEIR model parameters, the time-varying rate of infection, was estimated. Similarly, Li et al. [11] used the Ensemble Adjustment Kalman Filter (EAKF) on the augmented state-parameter space to estimate a modified deterministic SEIR model’s parameters. The augmented state-parameter can cause the EnKF to fail due to a strong nonlinear relation between the model parameters and its state [29, 30]. To overcome this, Engbert et al. [21] adopted a two-stage approach where the EnKF was initially used to estimate the states, followed by the likelihood-based inference of one of the SEIR model parameters.

Arroyo-Marioli et al. [25] applied the Kalman filter to estimate the time-varying growth rate of the COVID-19 cases. This was followed by an estimation of the time-varying effective reproduction number of the coronavirus disease. Finally, the time-varying effective reproduction number and disease transmission rate were employed by the SIR model in tracking the dynamics of COVID-19. Furthermore, Ghostine et al. [26] demonstrated the effectiveness of a joint-EnKF based assimilation scheme in estimating eight constant parameters of an extended SEIR model using the COVID-19 data.

The entire population is vulnerable to the disease at the first level of the outbreak. However, fewer individuals of size *S* are susceptible through control measures such as restriction of movements, self-isolation, and social distancing [31, 32]. While the initial number of susceptible individuals, *S*(0), is required for inverse modelling, estimating the actual population size under study can be challenging [31, 33, 34]. In the recent studies considering inverse COVID-19 modelling, several assumptions or methods were used to choose the total population size, *N*. These include assuming a fixed value for *N*, e.g. population of a city or a country [3, 7, 9–12, 14, 15, 21] or using a normalised version of a compartment model [2, 18, 20].

During the COVID-19 pandemic, there was more control of individual movements due to restrictions imposed by various governments. The restrictions included lockdown of cities, social distancing and quarantine measures. Other preventive measures were hand sanitation and wearing face masks. Considering the implementation of restrictions and preventive measures, in this study, we assumed that the infection rate, recovery rate and death rates of COVID-19 cases were all time-dependent. Estimating time-varying parameters of a model can be challenging, and the inverse problem may demand richer models [25].

Our contribution in this work includes evaluating the ensemble Kalman filter’s capability to estimate the time-varying parameters of the SIRD model. To overcome any challenges with the estimation of time-varying parameters, we used the EnKF with an augmented state-parameter scheme. To mitigate the problem associated with the nonlinearity between parameters and state, we tested the efficiency of the EnKF with different values of the damping factor [30, 35]. Additionally, we provide the EnKF algorithm to estimate the time-varying parameters.

The proposed method is demonstrated with test cases using synthetic data and the real COVID-19 data of Hubei province, China. There were some outliers in the reported number of cumulative cases of COVID-19 in the Hubei due to the change in diagnosing and revision of the definition of COVID-19 cases by the National Health Commission of the People’s Republic of China [3, 25, 36–38]. The time-varying model parameters were estimated using both the reported data of cumulative cases of COVID-19 in the Hubei province and for data consistency, using systematically modified data after removing the outliers.

The rest of the paper is structured as follows. We review the SIRD model and the ensemble Kalman filter algorithm for inverse modelling. Firstly, we demonstrate the use of EnKF using synthetic data. The effect on the estimated parameters using EnKF with different damping constant is illustrated with numerical simulations results using the synthetic case. Secondly, we show the usefulness of EnKF using the real COVID-19 data of Hubei province, China. Finally, we discuss the test cases results and end with the conclusion.

## Materials and methods

### Mathematical model

SIRD (Susceptible, Infectious, Recovered, and Dead) is a four-compartment model that has been widely used as a forecasting method of infectious disease [1–4, 16, 17, 22, 34]. In the SIRD model, the number of susceptible individuals (*S*), infected individuals (*I*), recovered individuals (*R*), and dead individuals (*D*) vary with time (*t*) as follows [2, 16, 17]:

$$\begin{array}{c} \begin{array}{cc} \frac{dS}{dt} & =\frac{-\beta }{N}IS\\ \frac{dI}{dt} & =\frac{\beta }{N}IS-\gamma I-\delta I\\ \frac{dR}{dt} & =\gamma I\\ \frac{dD}{dt} & =\delta I \end{array} \end{array}$$ (1)

where *β* is the transmission rate (infection), *γ* is the recovery rate, and *δ* is the death rate. The model estimation using COVID-19 data employs data on diagnosed cases. Hence, in that empirical context, *δ* can be better considered as the case fatality rate. Following Calafiore et al. [16] and Ianni and Rossi [34], *N* is defined as the fraction of the total population size that is affected by the contagion.

The model assumes that each individual who has already been infected can transmit the virus to those susceptible. Furthermore, the time length is considered short so that births and deaths not related to the virus are neglected. The SIRD model does not consider the effects of exposure, quarantine, confinement, or an asymptomatic population. This model is suitable for the case without any protection measures and restriction on activities, e.g. wearing masks and lockdown measures [22]. The capability of the simple SIRD model to capture the dynamics of COVID-19 has been demonstrated by Fanelli and Piazza [39] and Anastassopoulou et al. [3].

In the model, *N*(*t*) = *S*(*t*) + *I*(*t*) + *R*(*t*) + *D*(*t*) which is assumed constant [1, 2, 34]. The solution of the system of ODE depends on the initial conditions *S*_0_ = *S*(0), *I*_0_ = *I*(0), *R*_0_ = *R*(0), and *D*_0_ = *D*(0) for the susceptible, infected, recovered, and death populations, respectively. However, the initial number of susceptible population, *S*(0) = *N*(0) − *I*(0) − *R*(0) − *D*(0), is usually unknown since *N*_0_ = *N*(0) is typically unknown [16, 34]. This study assumes that the entire population, i.e. Hubei province, is vulnerable to the disease at the first level of the outbreak, and we let *N* = *N*(*t*) = *N*(0). However, it is noted that this number can be influenced by several factors such as geographical, social, and economic characteristics of the region under study.

Factors such as restricted movements of individuals, lockdown of cities, social distancing, quarantine measures, and preventive measures such as hand sanitation and wearing face masks allow the transmission rate to vary over time [25, 40, 41]. Hence, the transmission, recovery and death rates were all allowed to be time-dependent in this study. Similar to Avila-Ponce de León et al. [40] and Gupta et al. [41], the three time-varying parameters were defined as follows:

*The infection rate*: the time-varying infection rate before and after lockdown is described by

$$\begin{array}{c} \beta \left(t\right)=\{\begin{array}{cc} \beta_{0}, & t<t_{lockdown}\\ \beta_{0}\;\text{exp}(-\frac{t-t_{lockdown}}{\tau_{\beta }})+\beta_{1}, & t\geq t_{lockdown}. \end{array} \end{array}$$ (2)

*β*(*t*) is a function of three characteristic constants *β*_0_, *β*_1_ and *τ*_*β*_. Before lockdown, *β*(*t*) = *β*_0_ is a constant. When the lockdown is imposed at time *t* = *t*_*lockdown*_, *β*(*t*) decreases exponentially from *β*_0_+*β*_1_ to the final value *β*_1_ with a characteristic time of decrease *τ*_*β*_.

*The recovery rate*: With a new disease such as COVID-19, the health care system and medical staff have to learn and adopt new therapeutic procedures, including treatment of patients with new symptoms [40, 41]. Hence, the recovery time for patients may change with time. In this study, *γ*(*t*) is described by the function

$$\begin{array}{c} \gamma \left(t\right)=\gamma_{0}+\frac{\gamma_{1}}{1+\text{exp}(-t+\gamma_{\tau })}, \end{array}$$ (3)

where *γ*_0_ is the initial rate of recovery, and after *t* = *γ*_*τ*_ the final recovery rate becomes *γ*_0_+*γ*_1_.

*The death rate*: the death rate may also decrease with time due to factors such as adaptation of the pathogen and development of advanced treatments and vaccinations, including non-pharmaceutical interventions such as social distancing, lockdown of cities and increase in public awareness about the disease [40, 41]. The death rate *δ*(*t*) is described using the function

$$\begin{array}{c} \delta \left(t\right)=\{\begin{array}{cc} \delta_{0}, & t<t_{lockdown}\\ \delta_{0}\;\text{exp}(-\frac{t-t_{lockdown}}{\tau_{\delta }})+\delta_{1}, & t\geq t_{lockdown}, \end{array} \end{array}$$ (4)

where at time *t* = *t*_*lockdown*_, *δ*_0_ + *δ*_1_ is the initial death rate that decreases exponentially to the final value *δ*_1_ with a characteristic time of decrease *τ*_*δ*_.

For the SIRD model to simulate a particular epidemic with the three time-varying parameters, the nine characteristic constants (*β*_0_, *β*_1_, *τ*_*β*_, *γ*_0_, *γ*_1_, *τ*_*γ*_, *δ*_0_, *δ*_1_, *τ*_*δ*_), need to be estimated via inverse modelling.

### The ensemble Kalman filter for parameter estimation

Evensen [27] introduced the ensemble Kalman filter (EnKF), an algorithm for sequential data assimilation problems. Several papers are available for the derivation of the ensemble Kalman Filter (EnKF), including its algorithm, e.g. [42–45]. An ensemble of states is employed to approximate forecast states statistical information, including the model covariance matrix. The states are estimated by assimilating observations into the model in accordance with the Kalman filter formula [45]. The EnKF can be further adapted to estimate both model states and the unknown parameters using an augmented state-parameter scheme [26, 44, 46]. The steps of the augmented EnKF are summarized below [26, 44, 46].

Consider a discrete nonlinear model:

$$\begin{array}{c} s_{k+1}=S(s_{k},\theta_{k})+w_{k+1}, \end{array}$$ (5)

$$\begin{array}{c} \theta_{k+1}=\theta_{k}, \end{array}$$ (6)

$$\begin{array}{c} y_{k+1}=M\left(s_{k+1},\theta_{k+1}\right)+e_{k+1} \end{array}$$ (7)

where **s**_*k*_ = [*S*_*k*_ *I*_*k*_ *R*_*k*_ *D*_*k*_] is the vector of the state variables at time *t* = *k*, S is the nonlinear operator (SIRD model (1)), $\theta_{k}=[\beta_{0_{k}},\beta_{1_{k}},\tau_{\beta_{k}},\gamma_{0_{k}},\gamma_{1_{k}},\tau_{\gamma_{k}},\delta_{0_{k}},\delta_{1_{k}},\tau_{\delta_{k}}]$ is the vector of parameters that are assumed to remain constant in time, **w**_*k*+1_ is the model noise that is assumed to follow zero-mean Gaussian noise with covariance matrix **Q**_*k*+1_, **y**_*k*+1_ is the vector of observation (active number of infected cases, cumulative number of recovered cases, and cumulative number of death cases), M is the observation operator which connects the observed values to the state values of the model and **e**_*k*+1_ is the observation noise that is assumed to follow zero-mean Gaussian noise with covariance matrix **R**_*k*+1_.

At the forecast step, state variables, **s**_*k*+1_, and parameters, *θ*_*k*+1_, are augmented to form a vector

$$\begin{array}{cc} x_{k+1} & =[\begin{array}{c} s_{k+1}\\ \theta_{k+1} \end{array}]. \end{array}$$ (8)

For an ensemble of size *n*, an initial forecast ensemble of augmented vectors $X_{k+1}=[x_{k+1}^{{\text{f}}_{1}},x_{k+1}^{{\text{f}}_{2}},\ldots ,x_{k+1}^{{\text{f}}_{n}}]$ at *t* = *k*+1 is assumed known. The superscript f_*i*_ for *i* = 1, 2, …, *n* is the *i*^th^ forecast member of the ensemble **X**. Each *i*^th^ member of the ensemble is used to generate *i*^th^ realization of the model state vector using the forward model S. A set of corresponding measurement vector, $Y_{k+1}=[y_{k+1}^{{\text{f}}_{1}},\ldots ,y_{k+1}^{{\text{f}}_{n}}]$, is then generated where $y_{k+1}^{{\text{f}}_{i}}=M\left(x_{k+1}^{{\text{f}}_{i}}\right)\in R^{p}$. *p* denotes the number of observations at *t* = *k* + 1, and in this study *p* = 3.

In the analysis (assimilation) step, a perturbed observation vector, ${\hat{y}}_{k+1}^{i}={\hat{y}}_{k+1}+e_{k+1}^{i}$, for each *i*^th^ member of the ensemble is obtained using the current available observed data ${\hat{y}}_{k+1}\in R^{p}$. The random perturbations $e_{k+1}^{i}∼N\left(0,R_{k+1}\right)$ [46]. The *i*^th^ forecast member of **X**_*k*+1_ is then updated using the difference between perturbed observations and measurements according to:

$$\begin{array}{c} x_{k+1}^{{\text{a}}_{i}}=x_{k+1}^{{\text{f}}_{i}}+K_{k+1}[{\hat{y}}_{k+1}^{i}-y_{k+1}^{{\text{f}}_{i}}],\;\;i=1,\ldots ,n, \end{array}$$ (9)

where $x_{k+1}^{{\text{a}}_{i}}$ represents the *i*^th^ analyzed (updated) member of **X**_*k*+1_ and **K**_*k*+1_ is the Kalman gain matrix calculated as

$$\begin{array}{c} K_{k+1}=C_{{\text{xy}}_{k+1}}^{\text{f}}{\left(C_{{\text{yy}}_{k+1}}^{\text{f}}+R_{k+1}\right)}^{-1}. \end{array}$$ (10)

In Eq (10), **R**_*k*+1_ is the observation covariance matrix, and the covariance matrices $C_{{\text{xy}}_{k+1}}^{\text{f}}$ and $C_{{\text{yy}}_{k+1}}^{\text{f}}$ are defined as [45]:

$$\begin{array}{c} \begin{array}{c} C_{{\text{xy}}_{k+1}}^{\text{f}}=\frac{1}{n-1}\sum_{i=1}^{n}[x_{k+1}^{{\text{f}}_{i}}-{\bar{x}}_{k+1}^{\text{f}}]{\left[y_{k+1}^{{\text{f}}_{i}}-{\bar{y}}_{k+1}^{\text{f}}\right]}^{\text{T}},\\ C_{{\text{yy}}_{k+1}}^{\text{f}}=\frac{1}{n-1}\sum_{i=1}^{n}[y_{k+1}^{{\text{f}}_{i}}-{\bar{y}}_{k+1}^{\text{f}}]{\left[y_{k+1}^{{\text{f}}_{i}}-{\bar{y}}_{k+1}^{\text{f}}\right]}^{\text{T}}. \end{array} \end{array}$$ (11)

where ${\bar{x}}_{k+1}^{\text{f}}=\frac{1}{n}\sum_{i=1}^{n}x_{k+1}^{{\text{f}}_{i}}$ and ${\bar{y}}_{k+1}^{\text{f}}=\frac{1}{n}\sum_{i=1}^{n}y_{k+1}^{{\text{f}}_{i}}$ represent the ensemble averages.

At each *j*^th^ EnKF iteration, each member of **X**_*k*+1_, i.e $x_{k+1}^{{\text{f}}_{i}}$ for *i* = 1, …, *n*, is updated by assimilating perturbed observations using Eq (9). Henceforth, one EnKF iteration corresponds to one assimilation cycle. The procedure is iterated with the updated ensemble until a user-defined stop criterion is met, e.g. stopping criteria based on the maximum number of iterations or setting a threshold of the change of parameter values between two consecutive EnKF iterations. After the final iteration, the average of the ensemble is taken as the best estimate of the states and the unknown parameters, and the spread of the ensemble as the error variance [47].

### Damped-EnKF and convergence

The nonlinear relations between the model parameters and the measurements can cause the ensemble variance of parameters to collapse after a few cycles during the update step, leading to filter inbreeding (divergence) [30, 48]. In previous studies, Hendricks Franssen and Kinzelbach [30] and Rasmussen et al. [35, 49] showed that using a damping factor mitigates filter inbreeding and improves the parameter update in the assimilation step. The damping factor in the update step reduces spurious covariance resulting from an abrupt update of parameters [28, 30]. By applying a damping factor, *α*, in Eq (9), the *i*^th^ member of the ensemble is updated using

$$\begin{array}{c} x_{k+1}^{{\text{a}}_{i}}=x_{k+1}^{{\text{f}}_{i}}+\alpha K_{k+1}[{\hat{y}}_{k+1}^{i}-y_{k+1}^{{\text{f}}_{i}}],\;\;i=1,\ldots ,n. \end{array}$$ (12)

0 ≤ *α* ≤ 1 where *α* = 0 means no update of parameters during the assimilation step, and *α* = 1 means the basic scenario without any damping effect. In this study, the damping factor is only applied on parameter updates keeping the states updates undamped. To evaluate the influence of the damping factor on the performance of EnKF, different scenarios with *α* = 0.1 to 1.0, in step size of 0.1, were explored in this study.

In this study, the EnKF iterations (assimilation steps) are repeated until the following convergence criterion is satisfied:

$$\begin{array}{c} |\frac{\theta_{k+1}-\theta_{k}}{\theta_{k}}|\leq \text{tol}=0.001. \end{array}$$ (13)

The complete procedure for estimating uncertain and unknown model parameters using the ensemble Kalman filter is summarized in Algorithm 1.

**Algorithm 1** Augmented EnKF for estimation of model parameters

1: **Initialize:**

 *n* = No. of ensemble members

 Convergence *tol* = 0.001, *r* = 1

 **s** = [*SIRD*], *θ* = [*β*_0_, *β*_1_, *τ*_*β*_, *γ*_0_, *γ*_1_, *τ*_*γ*_, *δ*_0_, *δ*_1_, *τ*_*δ*_]

 **obs (time-series data)**: ${\hat{I}}_{k}$, ${\hat{R}}_{k}$, ${\hat{D}}_{k}$ for *k* = 0, 1, …, *t*_*obs*_

 **generate** an initial ensemble $x_{0}^{i}=\left[{}_{\theta_{0}^{i}}^{s_{0}^{i}}\right]$ for *i* = 1, …, *n*.

2: **while** *r* > *tol* **do**

3:  **for** *k* = 0 to *t*_*obs*_ **do**

4:   get observations: ${\hat{y}}_{k}=[{\hat{I}}_{k},{\hat{R}}_{k},{\hat{D}}_{k}]$

5:   set $R_{k}=\text{diag}[\sigma_{I}^{2},\sigma_{R}^{2},\sigma_{D}^{2}]$, where $\left(\sigma_{I},\sigma_{R},\sigma_{D})=0.1\times ({\hat{I}}_{k},{\hat{R}}_{k},{\hat{D}}_{k}\right)$

6:   **for** *i* = 1 to *n* **do**

7:    measurements: $y_{k}^{i}=M\left(x_{k}^{i}\right)$

8:    perturb observations: ${\hat{y}}_{k}^{i}={\hat{y}}_{k}+e_{k}^{i}$, $e_{k}^{i}∼N\left(0,R_{k}\right)$

9:   **end for**

10:   compute cross-covariance: $C_{{\text{xy}}_{k}}=\frac{1}{n-1}\sum_{i=1}^{n}[x_{k}^{i}-{\bar{x}}^{k}]{\left[y_{k}^{i}-{\bar{y}}^{k}\right]}^{\text{T}}$

11:   compute covariance: $C_{{\text{yy}}_{k}}=\frac{1}{n-1}\sum_{i=1}^{n}[y_{k}^{i}-{\bar{y}}^{k}]{\left[y_{k}^{i}-{\bar{y}}^{k}\right]}^{\text{T}}$

12:   compute Kalman gain: $K_{k}=C_{{\text{xy}}_{k}}{\left(C_{{\text{yy}}_{k}}+R_{k}\right)}^{-1}$.

13:   **for** *i* = 1 to *n* **do**

14:    assimilate (update): $x_{k}^{{\text{a}}_{i}}=x_{k}^{i}+\alpha K_{k}[{\hat{y}}_{k}^{i}-y_{k}^{i}]$

15:    $x_{k+1}^{i}\leftarrow x_{k}^{{\text{a}}_{i}}$

16:   **end for**

17:   convergence criterion: $r\leftarrow |\frac{\theta_{k}-\theta_{k-1}}{\theta_{k-1}}|$

18:   **end for**

19:  **end while**

20: **return** $x_{k+1}^{i}=\left[{}_{\theta_{k+1}^{i}}^{s_{k+1}^{i}}\right]$ and estimated parameter: ${\bar{\theta }}_{k+1}=\frac{1}{n}\sum_{i=1}^{n}\theta_{k+1}^{i}$

## Applications of EnKF in inverse modelling

The proposed damped-EnKF-based parameter estimation technique was applied to two test cases considering synthetic and real data. Firstly, we use the synthetic data to study the effect of different damping factors on the quality of the estimated parameters by the filter. This is followed by studying the sensitivity of the filter with different ensemble size. Finally, the EnKF with the selected damping factor and the ensemble size is used in the second test case using the real data.

### Parameter estimates with synthetic data

In the first test case, the performance of the EnKF was assessed using simulated data with synthetic observations. Table 1 shows the model parameters, $\theta_{k}=[\beta_{0_{k}},\beta_{1_{k}},\tau_{\beta_{k}},\gamma_{0_{k}},\gamma_{1_{k}},\tau_{\gamma_{k}},\delta_{0_{k}},\delta_{1_{k}},\tau_{\delta_{k}}]$, used to generate the synthetic data (observations). The model parameter values in Table 1 are referred as “true” (target) values. The system of ODE (Eq (1)) was solved numerically for, 0 ≤ *t* ≤ 100, with initial values *I*(0) = 350, *R*(0) = 1, *D*(0) = 7, and *S*(0) = *N* − 350 − 7 − 1, using MATLAB’s (version R2016a) ode45 solver. The population size *N* and *t*_*lockdown*_ were taken as 60M and 15, respectively. Synthetic observations were then recorded by extracting state values *I*(*t*), *R*(*t*) and *D*(*t*) representing the active number of infected cases, the cumulative number of recovered cases and the cumulative number of death cases.

**Table 1:** Parameters used in synthetic data generation.

Parameter	value	unit
*β*_0_	0.256	1/day
*β*_1_	0.001	1/day
*τ*_*β*_	14.39	day
*γ*_0_	0.017	1/day
*γ*_1_	0.06	1/day
*τ*_*γ*_	30.5	day
*δ*_0_	0.024	1/day
*δ*_1_	0.001	1/day
*τ*_*δ*_	21.6	day

The inverse problem involves employing EnKF to estimate the initially assumed true values of the parameters using synthetically generated observed values of *I*(*t*), *R*(*t*) and *D*(*t*). To study the effect of different damping factor on the filter’s performance, an ensemble of size *n* = 200 was chosen. The use of the EnKF with a 200-member ensemble has recently been shown to produce desirable results [26]. We use the state-parameter augmented EnKF, where *θ* = [*β*_0_, *β*_1_, *τ*_*β*_, *γ*_0_, *γ*_1_, *τ*_*γ*_, *δ*_0_, *δ*_1_, *τ*_*δ*_] and **s** = [*SIRD*], and the augmented vector is

$$\begin{array}{c} x_{0}=[s_{0}^{\text{T}}\;\;\theta_{0}^{\text{T}}]. \end{array}$$ (14)

Each state ensemble is initialised, for *i* = 1, 2 …, *n*, using normal distributions as follows:

$$\begin{array}{c} s_{0}^{i}=\text{max}[s_{0}(1+\mu \cdot \sigma ),0] \end{array}$$ (15)

where σ∼N(0,1) and *μ* is set to 20%. Initial ensemble for parameter values, for *i* = 1, 2 …, *n*, were randomly drawn from uniform distribution: ${\left(\beta_{0}^{i}\right)}_{0}∼U\left(0.2,0.6\right)$, ${\left(\beta_{1}^{i}\right)}_{0}∼U\left(0.05,0.15\right)$, ${\left(\tau_{\beta }^{i}\right)}_{0}∼U\left(10,30\right)$, ${\left(\gamma_{0}^{i}\right)}_{0}∼U\left(0.015,0.045\right)$, ${\left(\gamma_{1}^{i}\right)}_{0}∼U\left(0.02,0.06\right)$, ${\left(\tau_{\gamma }^{i}\right)}_{0}∼U\left(11,33\right)$, ${\left(\delta_{0}^{i}\right)}_{0}∼U\left(0.005,0.015\right)$, ${\left(\delta_{1}^{i}\right)}_{0}∼U\left(0.01,0.03\right)$ and ${\left(\tau_{\delta }^{i}\right)}_{0}∼U\left(12.5,37.5\right)$. The time span between two EnKF assimilation steps was taken as *dt* = 1*day*. Hence, the observations ${\hat{y}}_{t}\in R^{3}$, i.e. values of *I*(*t*), *R*(*t*) and *D*(*t*), were assumed to be known at *t* = 0, 1, …, 100.

In the assimilation step, perturbed observations are generated using ${\hat{y}}_{t}$. Therefore, the perturbed observations ${\hat{y}}_{k}^{i}={\hat{y}}_{k}+e_{k}^{i}$, for *i* = 1, 2, …, *n*. The additive noise $e_{k}^{i}∼N\left(0,R_{k}\right)$ where $R_{k}=\text{diag}[\sigma_{I}^{2},\sigma_{R}^{2},\sigma_{D}^{2}]$. *σ*_*I*_, *σ*_*R*_ and *σ*_*D*_ are the observation errors taken as 10% of the observed data values at time *t* = *k*. For convergence criteria, we set the tolerance value in Eq (13) as tol = 0.001. With the above setting, the proposed method was applied to retrieve the “target” (true) value of the model parameters using ten different damping constants. The accuracy of the proposed method was assessed by computing the Relative Mean Absolute Error (RMAE) of the simulated model state as

$$\begin{array}{c} \text{RMAE}=\frac{1}{N}\sum_{j=1}^{N}\frac{\left|y(j)-s(j)\right|}{\left|y(j)\right|}, \end{array}$$ (16)

where *y*(*j*) is the observed state, *s*(*j*) is the simulated state using the estimated parameters, and *N* is the sample size of the observed data.

**Fig 1:**
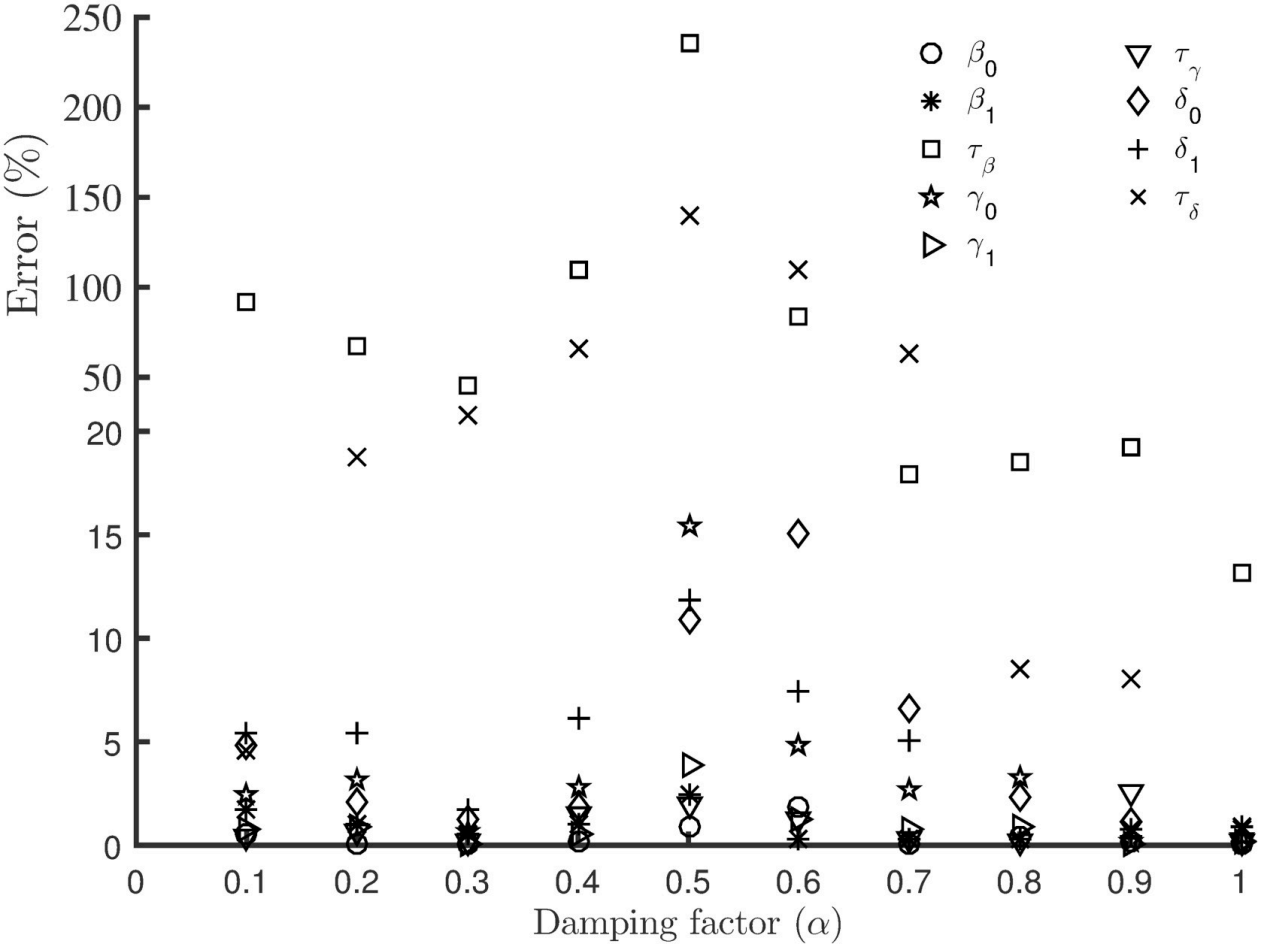
Comparison of errors in estimated parameters. The percentage error in the estimated parameters using EnKF (ensemble size, *n* = 200) with different damping factors.

**Fig 2:**
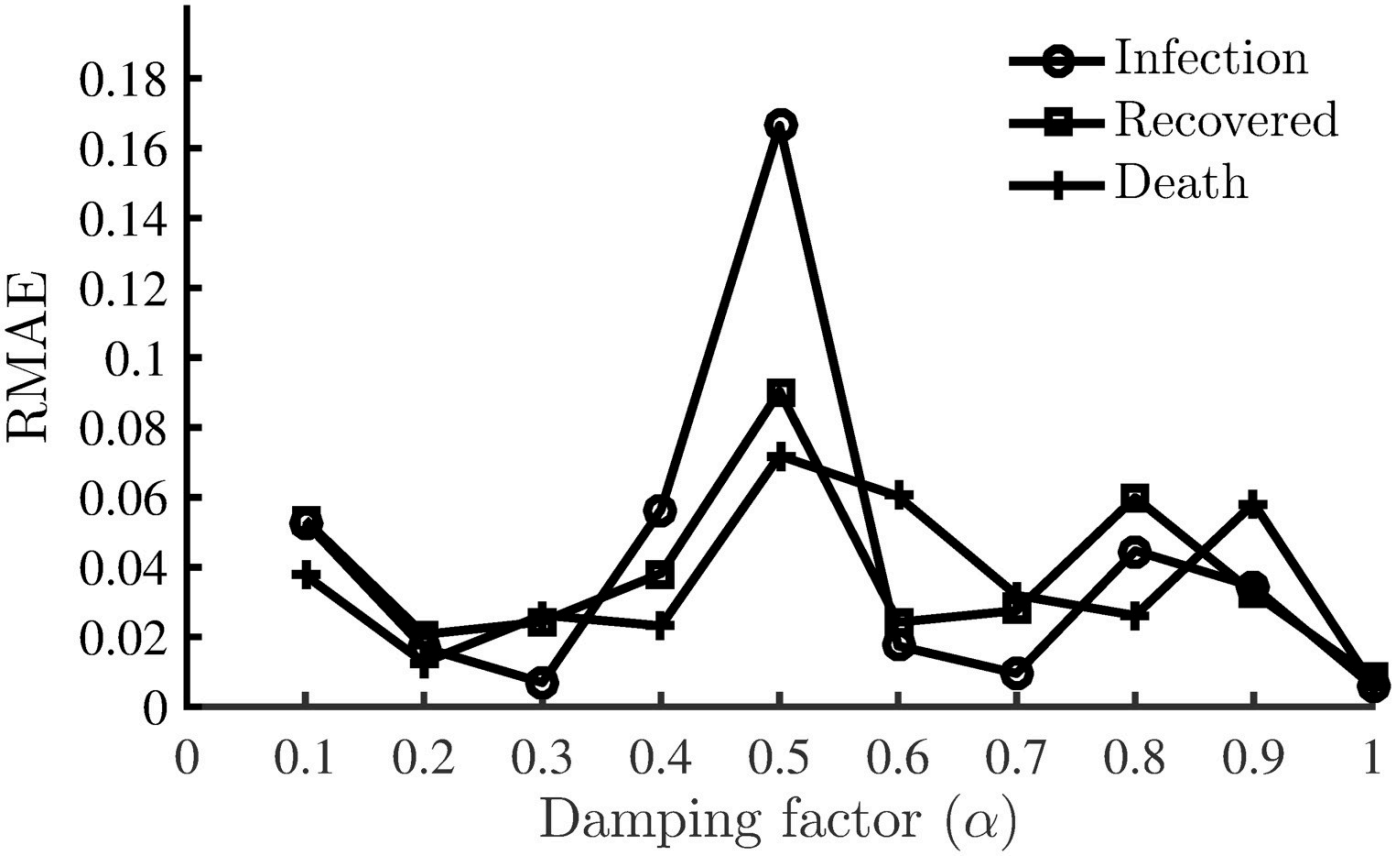
Comparison of RMAE with different damping factors. Relative Mean Absolute Error of the simulated model states (infected, recovered and death cases) as a function of damping factor.

Fig 1 shows the percentage error in the estimated parameters using EnKF (ensemble size, *n* = 200) with different damping factors. In this synthetic case, the best performance of EnKF is achieved by using the basic scenario without any damping effect, i.e. with *α* = 1. The most difficult parameter to estimate was *τ*_*β*_ that even with the basic scenario had an error of around 13% in the estimated value. Fig 2 shows the RMAE of the model states simulated using the EnKF estimated parameters with different damping factors. With the basic scenario, the computed RMAE’s was the least, with a value of less than 1%. The variability of the estimated parameters and RMAE with different damping factors led us to choose the EnKF with the basic scenario for the remaining test cases in this study.

**Fig 3:**
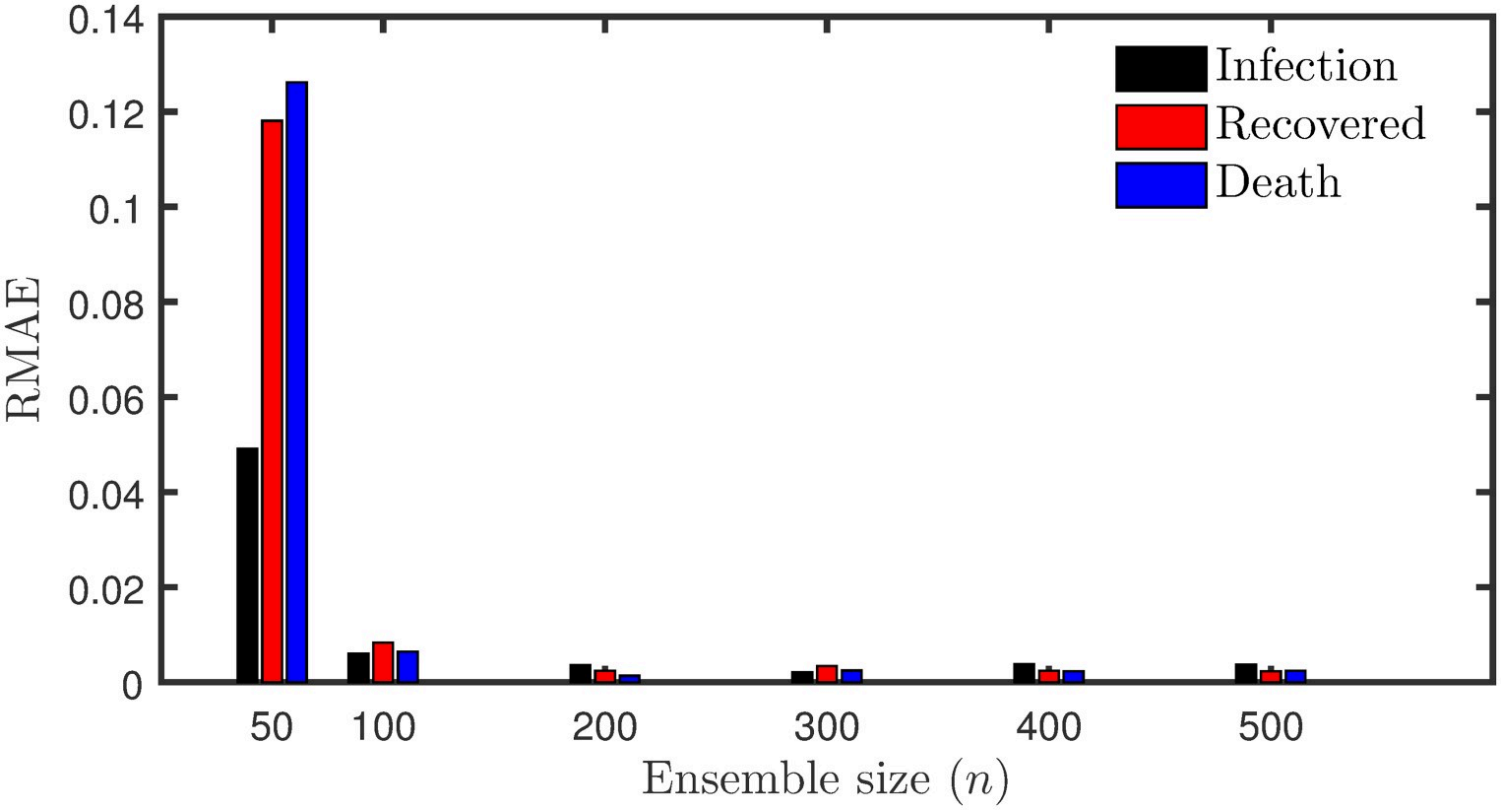
Comparison of RMAE with ensemble size. RMAE of the number of the infected, recovered, death cases as a function of ensemble size.

The sensitivity of the filter with different ensemble size is also studied. The augmented basic EnKF assimilation system was executed using six different ensemble sizes: *n* = 50, 100, 200, 300, 400, and 500. Fig 3 compares the RMAE of the simulated model states (infected, recovered and death cases) using the estimated parameters with different ensemble size. The result shows a considerable improvement in the performance by the filter beyond the ensemble size of 50. However, for *n* ≥ 200, there is not much improvement in the filter’s performance. For simplicity and computational cost reasons, an ensemble of size 200 is chosen for the rest of the test cases in this study.

**Fig 4:**
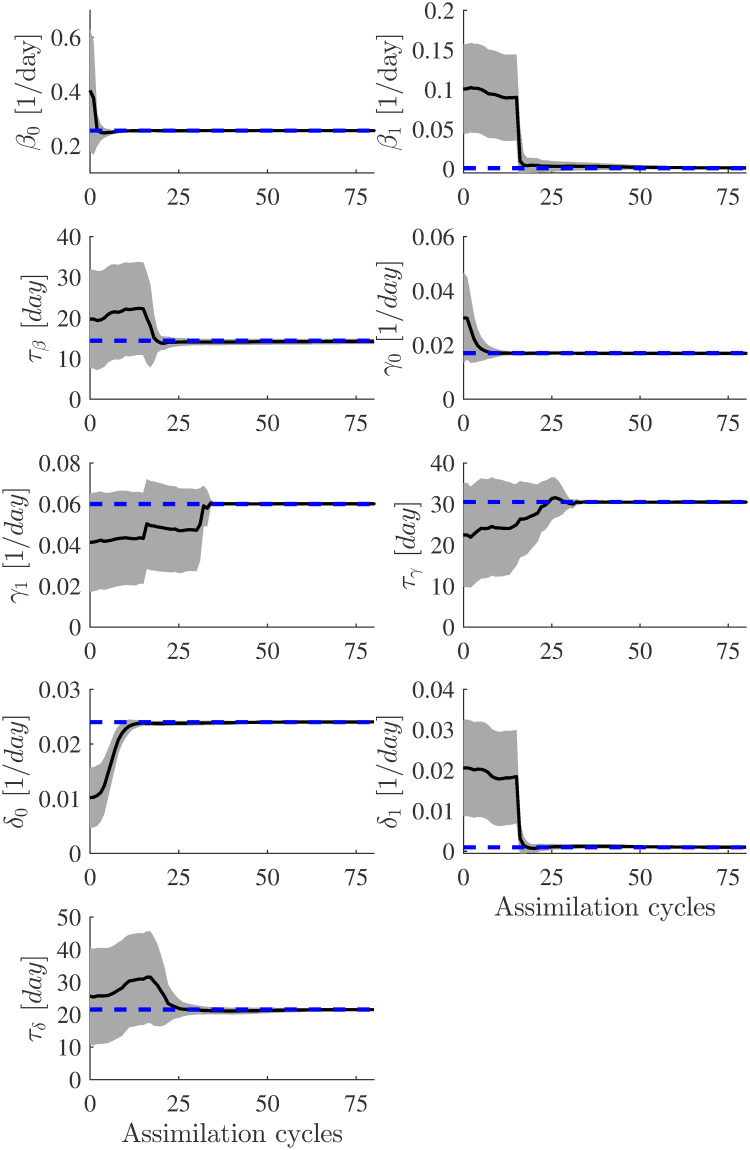
Evolution of the parameter estimates for the first test case (synthetic data). Estimated parameters using augmented EnKF (*α* = 1 and ensemble size *n* = 200). In each plot, the blue line represents the target value of the model parameters, and the solid black line represents the EnKF mean value. The uncertainties (standard deviation curves) around the mean values are filled in grey.

Fig 4 shows the estimated parameter evolutions using the basic augmented EnKF with *n* = 200. At the final EnKF iteration (assimilation cycles), the ensemble’s standard deviations around the average are considered the uncertainty (error) in the final estimate. Initially, the standard deviations around the mean are more significant, and as the parameters converge to the target values, they become tiny and are not easily visible in the plots. This suggests a high confidence level in the final estimates of the model parameters.

Table 2 presents the parameters estimated together with their associated uncertainties. All parameter estimates either converged to their target values or close to them. The parameter *τ*_*β*_ had the largest error (13%) in its estimated value. The results show that an ensemble of size *n* = 200 is sufficient to capture the true parameters.

**Table 2:** Parameter estimates for the first test case (synthetic data). EnKF estimated parameters with their associated uncertainties.

Parameter	Target value	Initial Uniform Range	EnKF estimate	% error in estimated value
*β*_0_	0.256	[0.2,0.6]	0.25597±0.00054	-0.01%
*β*_1_	0.001	[0.05,0.15]	0.00113±0.00048	13.00%
*τ*_*β*_	14.39	[10,30]	14.2672±0.59056	-0.85%
*γ*_0_	0.017	[0.015,0.045]	0.01692±0.00042	-0.47%
*γ*_1_	0.06	[0.02,0.06]	0.06008±0.00042	0.13%
*τ*_*γ*_	30.5	[11,33]	30.4511±0.11467	-0.12%
*δ*_0_	0.024	[0.005,0.015]	0.02403±0.00010	0.125%
*δ*_1_	0.001	[0.01,0.03]	0.00101±0.00078	1.00%
*τ*_*δ*_	21.6	[12.5,37.5]	21.5592±0.24538	-0.19%

**Fig 5:**
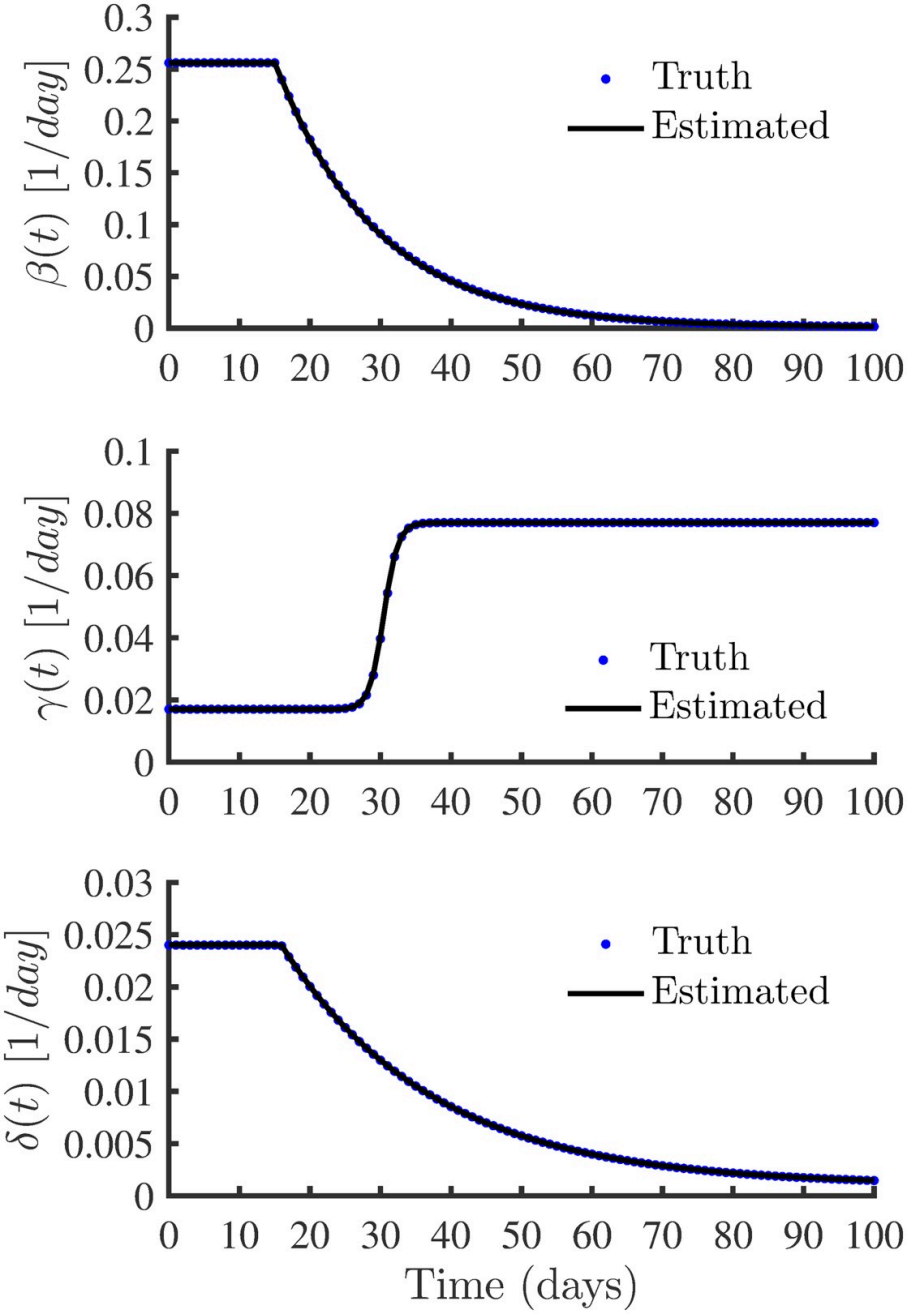
Comparison of time varying parameters. Best fit parameters of the time-varying infection, recovery and death rates.

**Fig 6:**
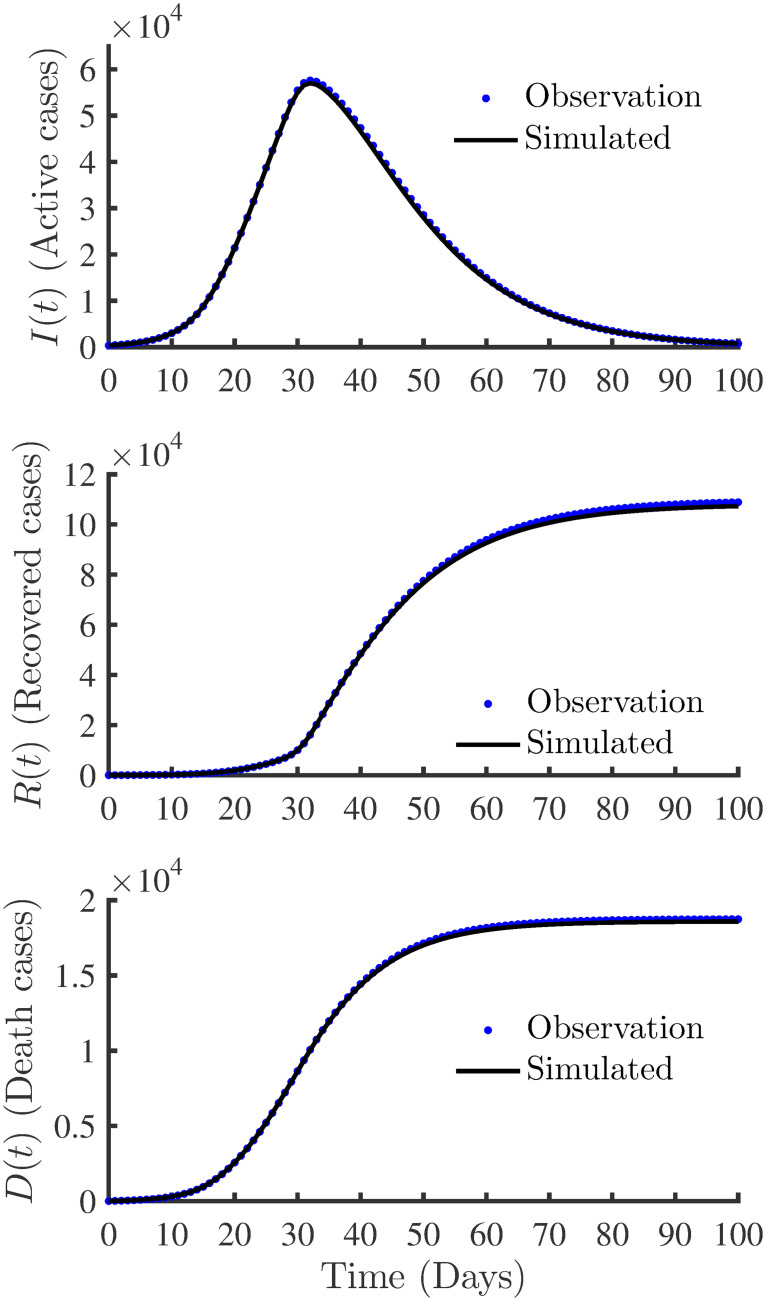
Profiles obtained using the SIRD model with true and estimated parameters. The plots show the accuracy of the curve fitting between the synthetic observations and the simulated profiles obtained with the estimated parameters.

Fig 5 shows the best-fit parameters of the time-varying infection, recovery and death rates. There is a good fit between synthetically generated (truth), and the model estimated variation of *β*(*t*), *γ*(*t*) and *δ*(*t*). Fig 6 shows the curve fitting accuracy between the observations and the simulated results of the SIRD model using the estimated parameters. There is a good fit of profiles indicating that the true model states are captured well through data assimilation using synthetic observations. In addition to computing RMAE to numerically quantify the accuracy and agreement between the observations and model-simulated results, the coefficient of determination, *R*^2^, values are computed using

$$\begin{array}{c} R^{2}=1-[\sum_{j=1}^{N}{\left(y(j)-s(j)\right)}^{2}/\sum_{j=1}^{N}{\left(y\left(j\right)-\bar{y}\right)}^{2}] \end{array}$$ (17)

where the variables *y*, *s* and *N* are as defined in Eq (16). Table 3 lists the RMAE and *R*^2^ values of the model states simulated using the EnKF estimated parameters. The RMAE values are less than 1% for recovered and death cases and less than 2% for active cases. Also, all *R*^2^ values are closer to 1, confirming a good quality fit of the simulated profiles with the observations.

**Table 3:** Performance of SIRD model with estimated parameters. RMAE and *R*^2^ values of the simulated states.

Indicator	Active cases (*I*)	Recovered cases (*R*)	Death cases (*D*)
RMAE (%)	1.44	0.96	0.54
*R* ^2^	0.9997	0.9995	0.9998

### Parameter estimates with real (COVID-19) data

#### COVID-19 data

In the second test case, the model parameters with their associated uncertainties were estimated using the reported COVID-19 data of Hubei province, China. Our analysis used the publicly available COVID-19 data from the GitHub repository by the Center for Systems Science and Engineering (CSSE) at Johns Hopkins University [50]. The repository provides time-series data for the cumulative number of confirmed cases *C*(*t*), the cumulative number of recovered cases *R*(*t*), and the cumulative number of death cases *D*(*t*). The data were double-checked against the reported statistics by the National Health Commission (NHC) of the People’s Republic of China (PRC) (http://www.nhc.gov.cn/xcs/yqtb/list_gzbd.shtml). The daily new confirmed COVID-19 cases data was taken from the reported data by NHC.

**Fig 7:**
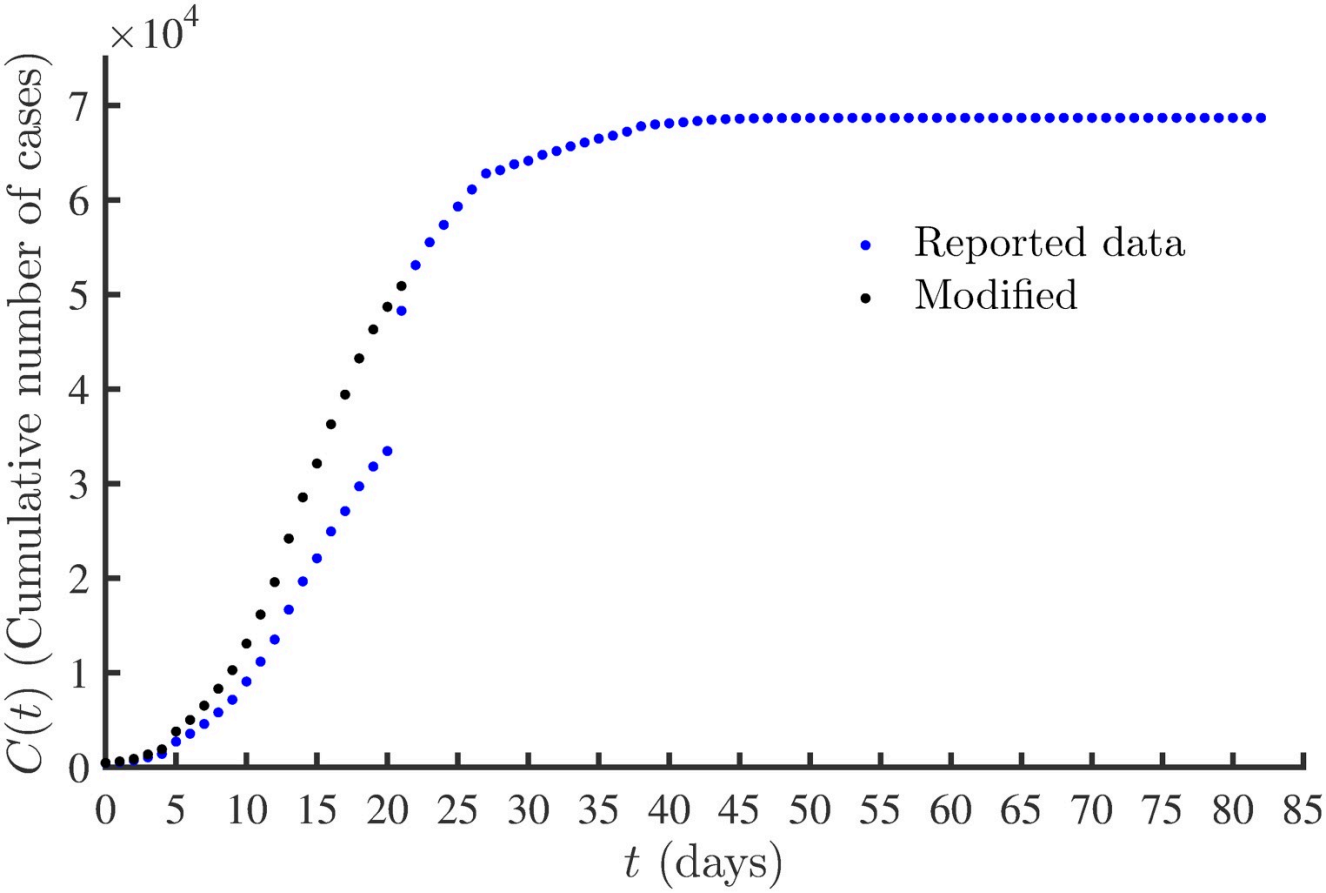
Cumulative number of COVID-19 cases. Cumulative number of COVID-19 cases in Hubei province, China. Blue dots represents the reported data and black dots represents the modified (reconstructed) time-series data from January 22, 2020 (*t* = 0) to April 13, 2020 (*t* = 82).

In December of 2019, the first case of COVID-19 was reported in Wuhan, Hubei province [5, 6]. The COVID-19 outbreak resulted in a restriction of individual’s movements in the city due to quarantine measures. The city of Wuhan was placed under lockdown beginning January 23, 2020, and the last city of Hubei province (Xiangyang city) was locked down on January 27, 2020 [7–9]. In our study, we used the time-series data from January 22, 2020 (*t* = 0) to April 13, 2020 (*t* = 82). Fig 7 shows the cumulative number of cases (blue dots) in the Hubei province from *t* = 0 to *t* = 82. On February 12, 2020, there was a surge in the reported number of new cases, as seen by the jump in the cumulative number of cases at *t* = 20 in Fig 7. The sudden increase of 14,840 new cases on February 12, 2020, was due to the change in diagnosis classification rule and revision of the definition of COVID-19 cases by the National Health Commission of the PRC [3, 25, 36–38].

To study the effect of data quality on the filter’s performance, the model parameters were estimated using both the reported data of cumulative cases and for data consistency, using systematically modified data. The modified data was obtained after removing the outliers and creating a new data series for the cumulative number of cases from the daily new cases. The reported 14,840 confirmed cases on February 12, 2020, included 13,332 clinical cases confirmed by the new diagnosis classification rule [51]. Recently, attempts have been made to remove such outliers from COVID-19 data and reconstruct a new time series from the number of new cases, e.g. Arroyo-Marioli et al. [25], Fu et al. [52], and Liu et al. [53].

We reconstructed the time-series data for the cumulative number of cases following the methods similar to that presented in Fu et al. [52] and Liu et al. [53]. It is noted that 4,823 new cases were reported on February 13, 2020. Compared to other days, this huge number of cases may also include cases that failed to meet the earlier diagnosis classification rule. For the number of new cases on February 12 and 13, we set it to 14,480 − 13,332 = 1,508. The extra 13,332 + 4,82 − 1,508 = 16,647 cases were added to the reported number of new cases (*NewCase*) from January 22 to February 14 in proportion to the original daily increment of the new cases. The new time-series data for the cumulative number of cases was obtained using *C*(*t*) = *C*(*t* − 1) + *NewCase*(*t*). The modified cumulative number of cases is shown with black dots in Fig 7. The number of infected (active) cases were then obtained using

$$\begin{array}{c} I(t)=C(t)-R(t)-D(t). \end{array}$$ (18)

#### Time-varying parameter estimation

As in the synthetic case, nine parameters describing the three time-varying model parameters, (*β*(*t*), *γ*(*t*) and *δ*(*t*)), were estimated using the observed time-series data of *I*(*t*), *R*(*t*) and *D*(*t*). To study the effect of the quality of the reported data on the filter’s performance, two models were estimated using different observed time-series data. Firstly, we used the values of *I*(*t*) obtained using Eq (18), where *C*(*t*) is the reported values. We refer to this as *case_orig*. Secondly, *I*(*t*) was obtained using the modified values of *C*(*t*), and we refer to this as *case_mod*.

The population size of the Hubei province was taken as *N* = 59*M* (https://data.stats.gov.cn/english/easyquery.htm?cn=E0103). For both, *case_orig* and *case_mod*, the initial state ensemble is generated using Eq (15) as in synthetic case with **s**_0_ = [*S*(0), *I*(0), *R*(0), *D*(0)], where *S*(0) = *N* − *I*(0) − *R*(0) − *D*(0) with *R*(0) = 28 and *D*(0) = 17 from the reported data. *I*(0) for *case_orig* and *case_mod* is 399 and 431, respectively. *t*_*lockdown*_ is set to 5. Similar to the synthetic case, the initial ensemble for parameter values was randomly drawn from a uniform distribution with the initial range of values presented in Table 4. All other EnKF parameters and settings were the same as for the synthetic case.

**Table 4:** Initial parameter values for *case_orig* and *case_mod*. Initial ensemble for parameter values randomly drawn from a uniform distribution with an initial range of values as presented below.

Parameter	Initial Uniform Range	Unit
*β*_0_	[0.1,0.9]	1/day
*β*_1_	[0.001,0.002]	1/day
*τ*_*β*_	[20,5]	day
*γ*_0_	[0.001,0.02]	1/day
*γ*_1_	[0.01,0.1]	1/day
*τ*_*γ*_	[40,7]	day
*δ*_0_	[0.001,0.0]	1/day
*δ*_1_	[0.001,0.002]	1/day
*τ*_*δ*_	[7,20]	day

**Fig 8:**
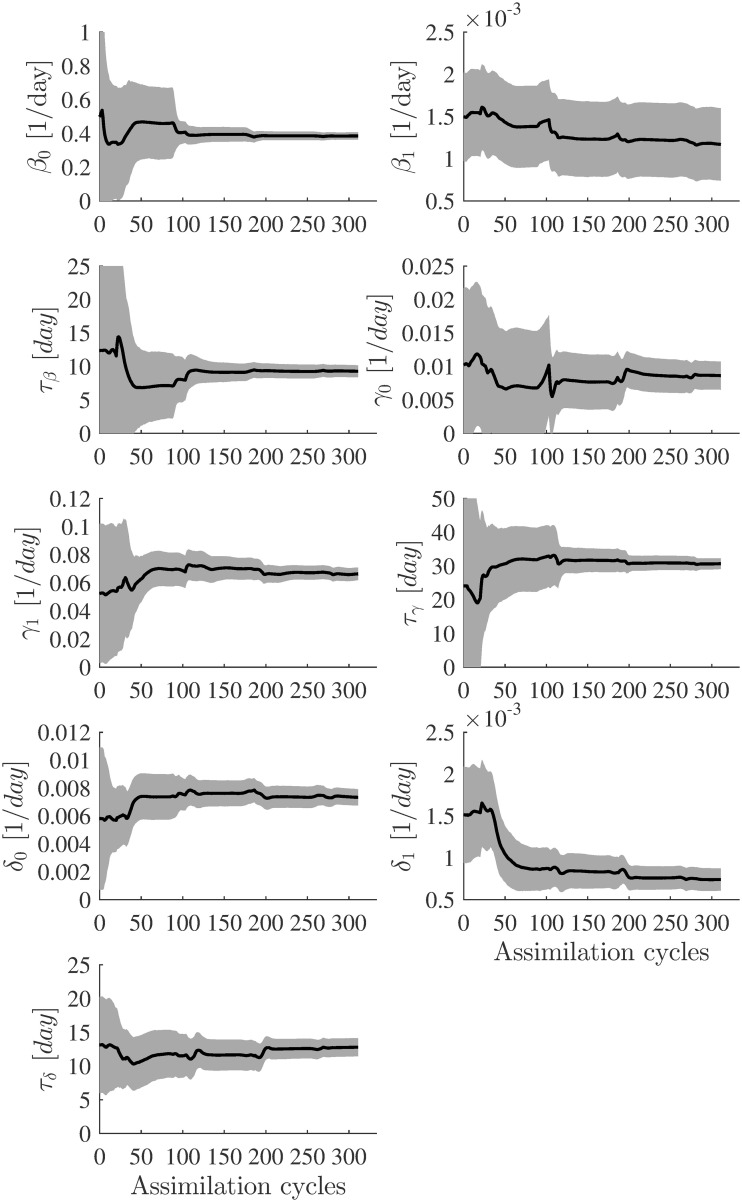
Evolution of the parameter estimates for *case_orig*. In each plot, the solid black line represents the EnKF mean value. The uncertainties (standard deviation curves) around the mean values are filled in grey.

**Fig 9:**
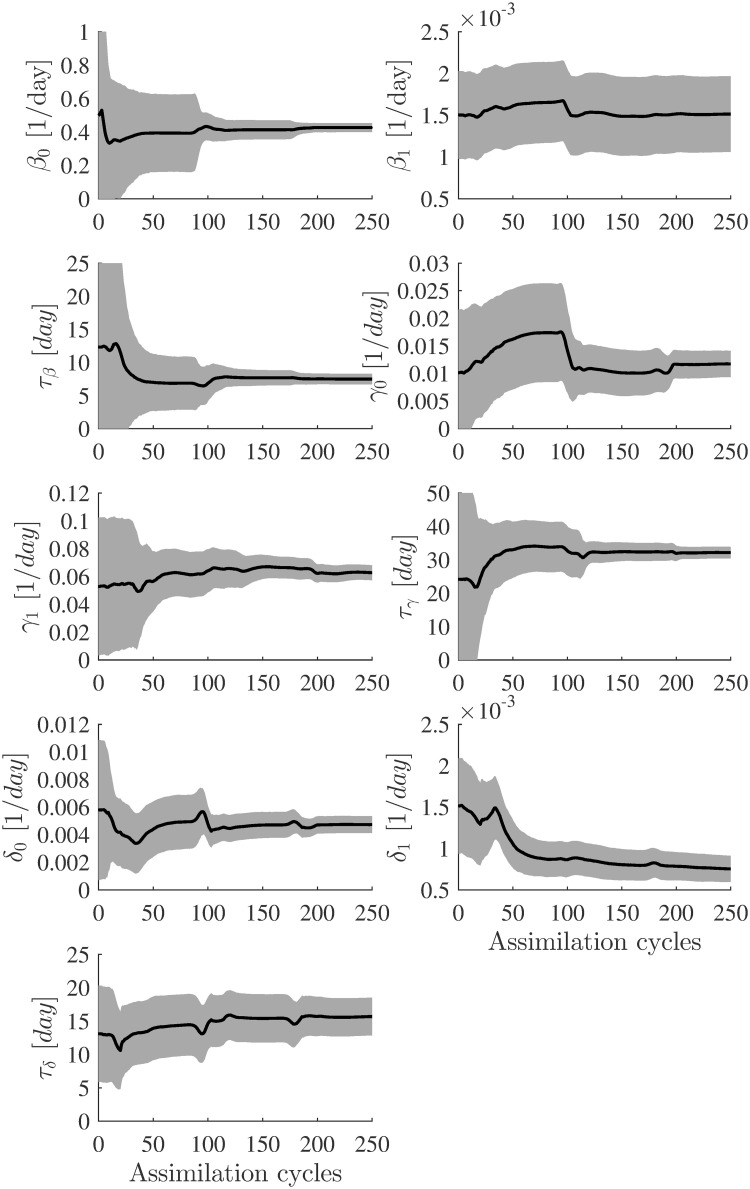
Evolution of the parameter estimates for *case_mod*. In each plot, the solid black line represents the EnKF mean value. The uncertainties (standard deviation curves) around the mean values are filled in grey.

The observed reported data of *I*(*t*), *R*(*t*) and *R*(*t*) are assimilated until the stopping criterion, Eq (13), is met. If the convergence criterion is not met once all observations are assimilated, the EnKF assimilation process is repeated with a different initial state ensemble. Figs 8 and 9 show the estimated parameter evolutions for *case_orig* and *case_mod*, respectively. The shaded areas show the uncertainties in the final estimate around the mean values. For *case_orig*, it took a long time to achieve convergence (333 assimilation cycles) compared to *case_mod*, which met convergence with 250 assimilation cycles. With *case_orig* and *case_mod*, the parameter *β*_1_ had the largest uncertainty in its final estimate. On the other hand, both cases had a small uncertainty in the estimation of *β*_0_.

**Fig 10:**
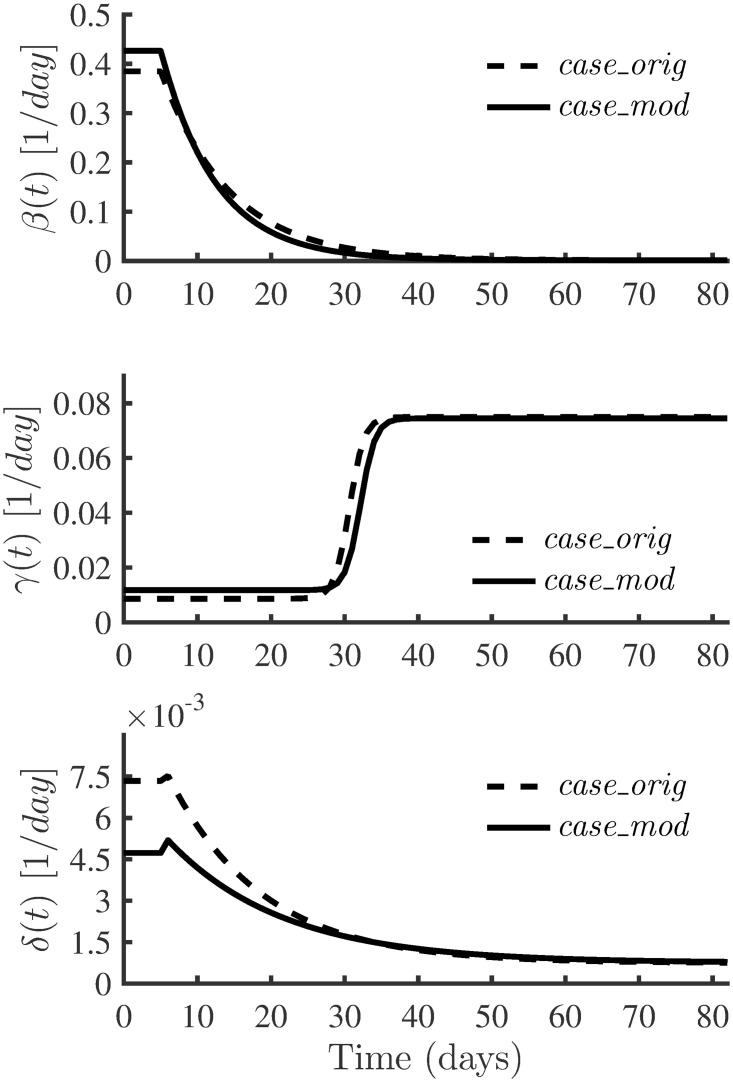
Comparison of time varying parameters with real data. The best-fit parameters of the estimated time-varying infection, recovery and death rates of *case_orig* and *case_mod*.

Table 5 presents the parameters estimated together with their associated uncertainties for *case_orig* and *case_mod*. The EnKF estimated different model parameters for *case_orig* and *case_mod*. Fig 10 compares the best-fit parameters of the estimated time-varying infection, recovery and death rates of *case_orig* and *case_mod*. Even though there are some differences between the estimated parameters from *case_orig* and *case_mod*, the estimated *β*(*t*) from the two cases show similar profiles. However, we notice some differences in the estimated *γ*(*t*) and *δ*(*t*) in the two cases between *t* = 0 and *t* = 25. A possible cause for this can be attributed to modifying time-series data in the same time range.

**Table 5:** Parameter estimates for the second test case (real data). EnKF estimated parameters with their associated uncertainties for *case_orig* and *case_mod*.

Parameter	unit	Estimated (*case_orig*)	Estimated (*case_mod*)
*β*_0_	1/*day*	0.3848±0.0107	0.4264±0.0133
*β*_1_	1/*day*	0.0012±0.0002	0.0015±0.0002
*τ*_*β*_	*day*	9.2746±0.4398	7.4757±0.3995
*γ*_0_	1/*day*	0.0086±0.001	0.0117±0.0012
*γ*_1_	1/*day*	0.0664±0.0023	0.0628±0.0027
*τ*_*γ*_	*day*	30.6556±0.7933	31.1524±0.8684
*δ*_0_	1/*day*	0.0073±0.0003	0.0047±0.0003
*δ*_1_	1/*day*	0.0007±0.0001	0.0008±0.0001
*τ*_*δ*_	*day*	12.7739±0.6605	16.3735±1.4076

**Fig 11:**
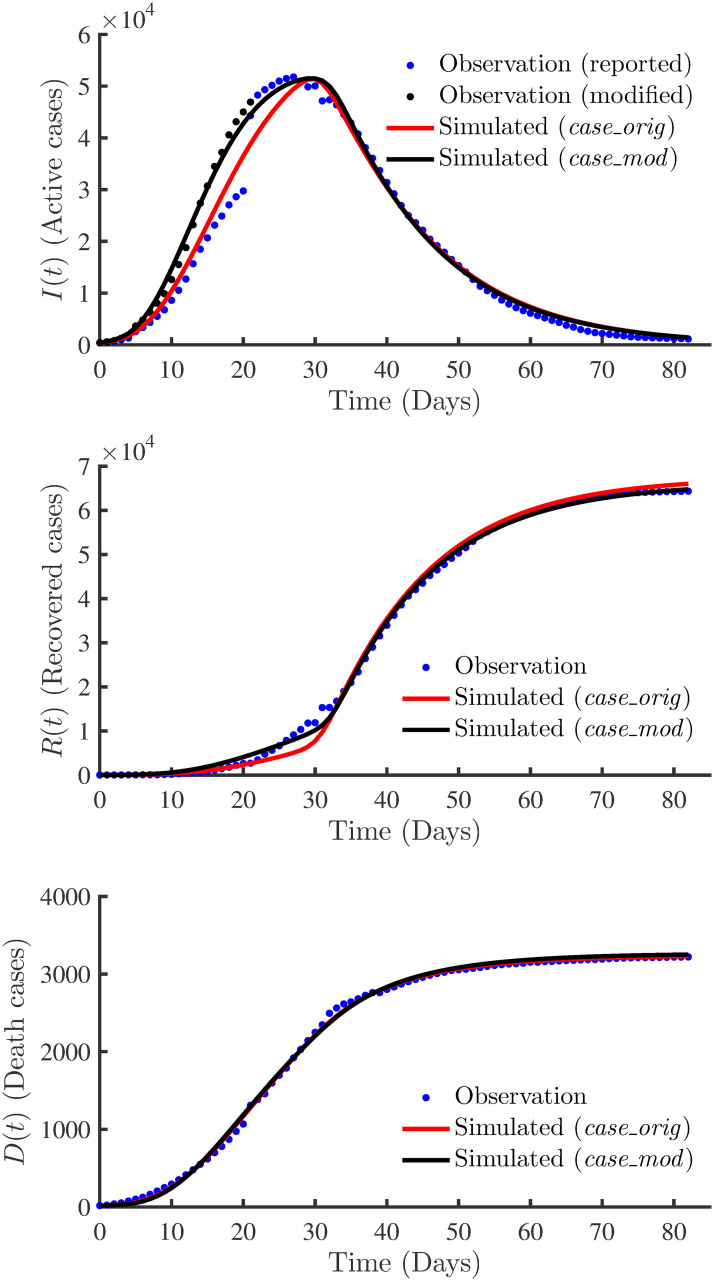
Comparison of profiles. The simulated profiles considering the estimated parameters for *case_orig* (in solid red lines) and *case_mod* (in solid black lines). The plots show the accuracy of the curve fitting between the simulated and observed data (blue dots for reported data and black dots for modified active cases).

Finally, in Fig 11, we show the curve fitting accuracy between the observations, i.e. reported *I*(*t*), *R*(*t*) and *D*(*t*), and the simulated results of the estimated model for *case_orig* and *case_mod*. We see a good fit for the infected population (active cases) for *case_mod*. However, there is a misfit with *case_orig* between *t* = 5 to *t* = 30. After *t* = 30, both the cases show similar profiles with a good fit. Likewise, in comparison with *case_orig*, *case_mod* shows a slightly better fit of the recovered population. However, both cases underestimate the recovered population between *t* = 20 to *t* = 30. Both cases well estimate the dead population. Overall, *case_orig* and *case_mod* show a good fit of the recovered and death populations, while case 1 shows an improvement in the estimation of the infected population. This is confirmed by the *R*^2^ values of the simulated states for the two cases, as presented in Table 6.

**Table 6:** Comparison of the estimated model using real data. *R*^2^ values of the simulated states for *case_orig* and *case_mod*.

Case	Active cases (*I*)	Recovered cases (*R*)	Death cases (*D*)
*case_orig*	0.9841	0.9963	0.9991
*case_mod*	0.9957	0.9987	0.9985

## Discussion

Recent works on the COVID-19 modelling using COVID-19 data of China include the works of Libotte et al. [18], Lobato et al. [2], Li et al. [11] and Cooper et al. [54]. To estimate the parameters using Stochastic Fractal Search (SFS) and Multiobjective Stochastic Fractal Search (MOSFS) algorithm, Lobato et al. [2] used a normalized version of the SIRD model using data of China. Similarly, Libotte et al. [18] used the normalized version of the SIR model and employed the Differential Evolution (DE) method to estimate the model parameters. Cooper et al. [54] estimated the SIR model via data fitting with a nonlinear function using COVID-19 data of China. Similarly, Li et al. [11] used an SEIR model based on deterministic assumptions and applied the EAKF to estimate model parameters using the data of China.

The transmission rate, *β*, is considered as an important parameter that needs to be estimated for epidemic modelling [29]. Table 7 presents the comparison between the EnKF estimated infection rate, *β*(0), with the estimated *β* from the recent works mentioned above. The values in Table 7 are directly taken from the reported results of five different methods (SFS, MOSFS, DE, EAKF and data fitting) presented in the reference literature [2, 11, 18, 54]. We observe that the EnKF estimated value of *β* = 0.3848 is very close to the values estimated from other methods in [2, 11, 18, 54]. This means that the infection rate was similar irrespective of the population size. In Fig 10, we see that the recovery rate from *case_orig* and *case_mod* is initially slower and later reaches a constant value of *β* ≈ 0.074, corresponding to a recovery time of ≈14 days. The estimated value of *β* agrees with the median recovery time of 2 weeks for mild COVID-19 cases as reported by the World Health Organisation [55].

**Table 7:** Comparison of transmission (infection) rate. Comparison of estimated *β*(0) from *case_orig* with some recent work. The values are directly taken from the reported results of five different methods (SFS, MOSFS, DE, EAKF and data fitting) presented in the reference literature [2, 11, 18, 54].

Method	Model	Country and duration (2020)	*β* *day* ^−1^
EnkF	SIRD	Hubei, China (Jan 22—Apr 13)	0.3848
SFS [2]	SIRD	China (Jan 22—Apr 2)	0.369
MOSFS [2]	SIRD	China (Jan 22—Apr 2)	0.377
DE [18]	SIR	China (Jan 22—Apr 2)	0.357
Data fitting [54]	SIR	China (Jan 01—Jun 30)	0.350
EAKF [11]	SEIR	China (Jan 24—Feb 8)	0.35

In this study, the best performance was achieved with a damping factor of *α* = 1. The EnKF method presented may not be the best method for estimating a basic SIR model and thus should be considered as an alternative for inverse modelling. Other more straightforward methods and optimization techniques such as least-square techniques [3, 10, 12, 14, 15], Differential Evolution method [18], and Stochastic and Multiobjective Fractal Search algorithm [2] can be employed. An advantage of using EnKF lies in the fact that it can provide a reliable uncertainty in the estimated parameter values. Hence, the EnKF makes it easier to quantify estimation uncertainty. Moreover, in comparison to other optimization methods, the observed data is assimilated in real-time with EnKF. EnKF is a derivative-free method in the sense that it does not require derivatives of the model function. This gives the EnKF an advantage over other optimization techniques that require derivatives, such as an extended Kalman filter. Hence, the EnKF can be used with any forward model, including complex and high dimensional models. However, the EnkF can be computationally demanding, especially with a larger ensemble size.

Our study’s obvious limitation is determining an optimal value of the damping factor for inverse modelling using the EnKF. It is important to emphasize that COVID-19 data of only Hubei province, China, was used in this study. Also, the inverse modelling was performed using the data after January 22, 2020, when there was more control of individual movements due to the Chinese government’s various restrictions. Further studies are warranted to find an optimal value of the damping factor. The one straightforward recommendation is to apply the proposed method using COVID-19 data of other countries to identify any similarities in the damping factor.

Two cases considering the real data, *case_orig* and *case_mod*, were used to study the performance of EnKF in terms of model estimation. Even though there was a slight difference between the estimated models from the two cases, one can apply a different procedure to remove the outliers from the reported data and obtain another time-series data for the infected (active) number of cases, e.g. one may adopt the method presented in Arroyo-Marioli et al. [25]. Moreover, different forms of time-varying parameters can be used in this study, e.g. Yang et al. [20] estimated the transmission (infection) rate, *β*(*t*), as a piecewise constant function in time while Arroyo-Marioli et al. [25] used a random walk model for *β*(*t*).

However, the results obtained from the use of the EnKF demonstrate its usefulness in estimating the unknown and uncertain parameters of an epidemic model. Moreover, the EnKF results show that it is possible to identify the time-varying model parameters with uncertain observational data.

## Conclusion

In this study, we evaluated an augmented Ensemble Kalman Filter’s capability to estimate time-varying model parameters using two types of observational data, i.e., synthetic data and with COVID-19 data of Hubei province, China. Furthermore, we investigated the effect of the damping factor on the performance of the EnKF. Three time-varying SIRD model parameters were determined by estimating nine constant parameters.

The best performance of EnKF was obtained using the basic EnKF scheme. Good performance was achieved with a small ensemble size of 200. The results presented in this study shows that epidemiological models can be estimated using EnKF even from imperfect data that can result from missing, incomplete or incorrect data. As an alternative to existing optimization techniques, one can use the EnKF algorithm presented in this paper to estimate uncertain and unknown model parameters with their associated uncertainties.
